# Determinants of blockchain adoption in news media platforms: A perspective from the Vietnamese press industry

**DOI:** 10.1016/j.heliyon.2022.e12747

**Published:** 2022-12-30

**Authors:** Chien Thang Pham, Trang Ta Thi Nguyet

**Affiliations:** a Faculty of Journalism and Communication, TNU-University of Sciences, Viet Nam; b Department of Economics and Management, TNU-International School, Viet Nam

**Keywords:** Blockchain, Behavioral intention, Journalism, Technology affinity, Regulatory support, UTAUT

## Abstract

Blockchain technology is being applied worldwide. Although a large body of blockchain research has been conducted in various fields, little is known about press perspectives on adopting blockchain in journalism. This study explores the determinants of applying blockchain in journalism activities in Vietnam. Based on Unified Theory of Acceptance and Use of Technology (UTAUT) and previous research, we surveyed 287 people working at press agencies in Vietnam. The results from testing nine research hypotheses show that five factors, namely Technology Affinity, Effort Expectancy, Facilitating Condition, Technology Readiness, and Regulatory Support, positively impact the intention of applying Blockchain in journalism activities in Vietnam. Two factors, namely Performance Expectancy and Trust, were not positively strongly correlated to the use of blockchain. Besides, Regulatory Support is found to have a moderating effect on the relationship between Facilitating Condition and Behavioral Intention.

## 1 Introduction

Blockchain (BC) is a distributed and secure data storage technology that enables a system to group data into “chains”, encapsulate it in small “blocks”, and record it in a strict block order [[1], [2], [3]]. BC technology is distinguished by decentralization, openness, autonomy, tamper resistance, anonymity, and high security. BC technology can bring about transparency and trust of information [4]. Also, this new journalism BC-based model can ensure the right to share information, process personalized journalism, and enhance the role and responsibility of journalists in the content production process [5]. As the potential benefits of blockchain for the media sector mainly involve payment transactions and copyright tracking, content creators may be able to monitor their playtime closely and royalty; as a result, advertising earnings might be distributed accurately, promptly, and at a low cost based on consumption [6].

In recent years, traditional journalism activities worldwide have encountered such problems as the spread of fake news, advertising frauds, difficulties in copyright management, poor-quality content, and challenges to press freedom. In addition, there remain fundamental shortcomings related to the mechanism of news content production, such as political impacts and unprotected interests of content creators, leading to low-quality content [7,8].

The contemporary literature shows that BC technology has been mentioned as an effective solution to the problems above [4,[8], [9], [10], [11], [12]]. For example, the use of BC technology can help news agencies safely keep track of the publication date and sources of news stories. For the advertising business, which journalism relies on, blockchains can store and keep track of advertising impressions so that media organizations do not need to pay too much to an advertisement that is not popular. Although BC technology is still in its early stages and is not commonly used in the media business, it demonstrates its global potential.

In Vietnam, the Government has approved the National Digital Transformation Project 2025–2030; the main goal is to digitalize more industries by testing new technologies and models by 2030 [13]. At the beginning of 2022, the Vietnam Blockchain Alliance under the Vietnam Digital Media Association was established and officially operated with an ultimate goal of developing BC technology in Vietnam [14]. In journalism, the Ministry of Information and Communications of Vietnam has also developed a Proposal for Strategic Digital Transformation of Newspapers 2025–2030, whose goal is to develop digital press products, digitalize press content, and improve the readership quality.

Although many studies have investigated the use of BC technology (e.g., Refs. [[15], [16], [17], [18]]), there has been no such research on the determinants of BC adoption in the field of journalism. This study explores the determinants of BC adoption in journalism in Vietnam. The research results will help modify the research model for the adoption of BC in the field of news media in developing journalism in Vietnam and similar contexts.

## 2 Literature review

### 2.1 Blockchain adoption in the field of journalism

**Fig. 1:**
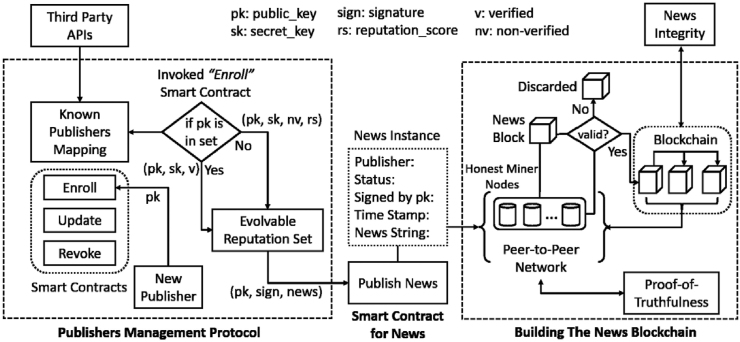
The proposed architecture of the blockchain-based news verification framework. *Note*: Adapted from “Using Blockchain to Rein in the New Post-Truth World and Check the Spread of Fake News” by Ref. [4]; *IT Professional*, p. 20.

The most anticipated adoption of BC is in the news media platform [6]. Based on current blockchain news platform activities, it is possible to broadly categorize the present BC technology usage in the news sector into two groups: fake news governance and the development of new news modes [8]. Contemporary literature shows that news contents need verifying because fake news, deliberate falsehoods generated and spread by traditional or social media, may make individuals misunderstand agendas. Seriously, it may intentionally misinterpret governments’ policies. Therefore, it has grave repercussions for our democracy, freedom, and social principles. Widespread hoaxes comprise dramatic, eye-catching, and manufactured information that is created purposely to deceive, hurt, and achieve specific political and financial goals [4,12].To solve the problem, data storage and validation techniques using BC data structures can play a better role in dynamic data management because it has tamper mechanisms to prevent access to confidential data, establishes a data source system to track and determine news sources, and protects the established system [12]. According to Qayum et al. (2019), as a decentralized ledger technology, BC technology promisingly brings transparency and trust to the news authentication and anti-fake news process [4] (see Fig. 1 [4,4,4,4]).

Online advertising is becoming more common, putting consumer privacy at risk [11]. Due to this, the British Columbia-developed digital advertising system has specific technological advantages over the existing one that can be used to address some of its shortcomings, including advertising fraud, unsecure user privacy data, difficulty tracking advertising effect, invasion by intermediaries, and unequal treatment of value chain stakeholders [8]. For instance, a BC advertising reporting system based on user behavior verification was developed by Stylianos and George (2017). Honest users would receive BC tokens after seeing an advertisement. Advertisers can easily spot shady activity because user feedback and tokens are sent as a blockchain.

Digital asset transactions can be carried out without having faith in other people or institutions thanks to the consensus mechanism of the blockchain and smart contracts, hence decreasing transaction costs and hazards. Therefore, BC-based copyright protection is viewed as a potential replacement for the current digital copyright protection system [19]. A potential new option to the current digital copyright protection is BC-based copyright protection [8,19]. A social media notarization method based in British Columbia was proposed by Ref. [20].

**Fig. 2:**
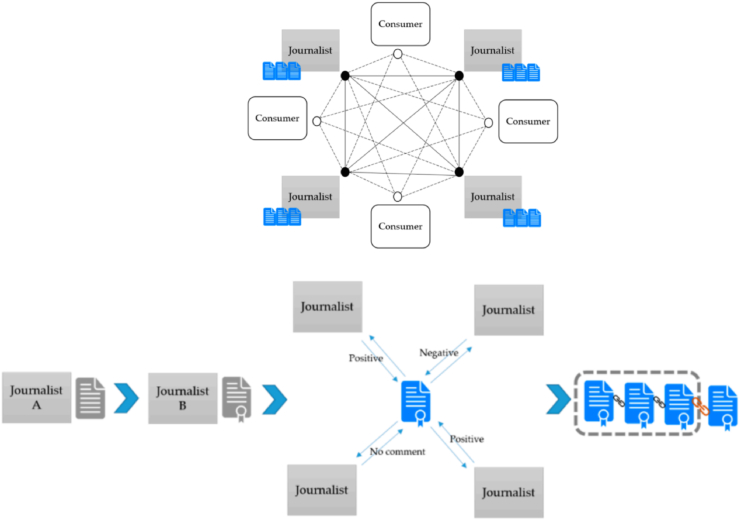
Distributed system by hybrid blockchain for journalism and The process of proof of sharing. *Note*: Adapted from “Journalism model based on blockchain with sharing space” by Kim B, Yoon Y., 2019, *Symmetry*, p. 7–8.

A BC content production platform's core features include establishing a new business model for content production, minimizing or doing away with the need for middlemen to connect content producers and consumers, streamlining the value chain, and giving content producers more control, quicker financial success, and a more flexible licensing model [21]. Journalists may produce unbiased material in the news ecosystem created by BC technology without being influenced by politics, finance, or technology, ensuring the neutrality of news output. Readers can consult with reporters to receive all the information they need, and reporters can speak directly with readers to learn about their actual information needs. Kim and Yoon [5] suggested a shared space news model that promotes restraint and self-discipline and is based in British Columbia. The platform will give journalists and users distinct permissions. In order to stop the propagation of false information, journalists should have the freedom to write and access the BC. On the platform, multiple groups may simultaneously monitor journalists (see Fig. 2).

Besides, for better quality, it is necessary to improve the role and benefits for journalists. Journalists can receive equal returns for their efforts [22]. BC technology establishes a new business model for content production, reducing or eliminating the need for intermediaries between journalists and the public; the new model can give content creators greater control over their work, more adaptable license agreements, and a larger share of content revenue, and quicker monetization. A lower role for intermediaries will result in more effective income distribution throughout the supply chain for publishing companies and others [21].

The abovementioned initiatives all demonstrate the growth trend of content production platforms supported by BC technology. Despite the fact that BC technology is still in development and is not yet extensively employed in journalism, it demonstrates the field's potential for usage.

### 2.2 Unified theory of technology adoption and use

To understand BC acceptance behavior and barriers to BC adoption, the UTAUT is used in studies on adopting and using technology across different sectors [23–26]. It is an influential model used in the adoption of new technologies, such as cloud computing [25] and mobile payments [24], promoting the adoption of BC in supply chain management in the United States of America and India [27], and similar to the supply chain in Malaysia [28] and Brazil [29], technology adoption in health care [30], and student behavior towards the use of mobile learning [31].

The UTAUT model has been widely used in research to explain and predict technology acceptance behavior. UTAUT was first proposed by Ref. [26]; which was based on several research models (theory of rational action, the technology acceptance model, the theory of planned behavior, the theory of innovation spillover). It considers the impact of four key factors, namely performance expectancy, effort expectancy, social influence, and facilitating conditions, on the intended behavior for technology adoption. The four included regulatory variables are gender, age, voluntariness, and experience [26,32,33].

The UTAUT model has been recently modified. Recent studies have added new factors from other theoretical models. Such factors as the suitable information technology infrastructure, technology readiness, and available human resources can enable companies to apply technology easily [34,35], Technology Affinity is also taken into account when the level of technology friendliness is considered an important factor to increase the possibility to adopt new technology [36,37], Trust is assessed as a major factor and directly affects an individual's adoption of new technology [29,38,39]. Regulatory Support is also regarded as an essential component in technology adoption due to the nation's role, policies, and related regulations that must be followed, all of which have varying effects on the adoption of technology in an organization's operations [28,40].

However, studies on the additional factors mentioned above have given controversial results. While the study by Ref. [28] indicated that Performance Expectancy, Effort Expectancy and Trust had no positive effect on the intention of adopting BC in supply chain management in Malaysia, the study by Ref. [27] showed that these were significant factors. Camerer et al. [41,42] argue for the need to test and retest the UTAUT model believe that it should be tested in different fields and contexts to modify this model.

## 3 Research hypotheses and models

**Fig. 3:**
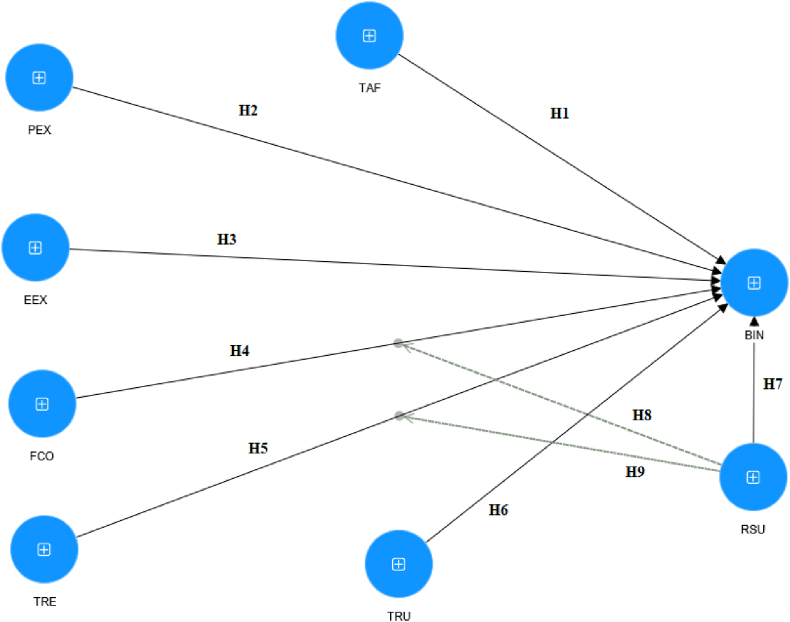
Research model.

In this study, the research model is based on UTAUT [26] with extended factors proposed by Refs. [28,43]. Accordingly, the effect of Regulatory Support is considered. However, such regulatory variables of the UTAUT model as gender, age, voluntariness, and experience are not included. Since BC adoption is still new in Vietnam, with unknown potential to many people, this study does not consider the assessment of the impact of Social Influence factors on the acceptance of BC use. Fig. 3 is a research model of the determinants of BC adoption in news communication activities of this study.

*Technology affinity* (TAF) is an individual's tendency to accept new technology. The impact of TAF on the intention of adopting new technology [28,36,37]. Those who have a positive attitude towards technology can be motivated to access new technology more efficiently [44]. In the field of journalism, technology-friendly media and journalists are able to learn relevant knowledge and apply technology more quickly. The hypothesis here is.

H1 TAF has a positive influence on Behavioral Intention (BIN) to apply BC in the field of Journalism in Vietnam.

*Performance Expectancy* (PEX) can be understood as an individual's expectation that adopting new technology is probably effective for their job [26]. Recent studies by Ref. [45]. Jung et al. and Venkatesh et al. [33,46] show that PEX influences technology adoption. However, Queiroz et al. and Wong et al. [28,29] argue that the adoption of BC in supply chain management may depend on the context. To find if this factor is a determinant in accepting BC in journalism in the Vietnamese context, we propose the hypothesis.

H2 PEX has a positive influence on BIN of applying BC in the field of Journalism in Vietnam.

*Effort Expectancy* (EEX) is the ease of adopting a new technology [33], which has been found to be a good predictor of technology adoption in an organization [25,26,29]. However, the study by Ref. [28] showed that EEX positively affect the intention of applying BC in supply chain management in Malaysia. Based on the aforesaid controversial results, we retest this relationship in the Vietnamese context with the hypothesis:

H3 EEX has a positive influence on BIN to apply BC in the field of Journalism in Vietnam.

*Facilitating Condition* (FCO) refers to an individual's perception of resources and support in adopting new technology. This refers to the extent to which an individual believes the technical infrastructure and organizational conditions are suitable for technology adoption. The current literature indicates that this is a good predictor of technology adoption in an organization [26,28,29,33]. Therefore, we propose the following hypothesis:

H4 FCO has a positive effect on BIN of applying BC in the field of journalism in Vietnam.

*Technology Readiness* (TRE) is known as the proper infrastructure of an organization for people to apply a new technology at work. TRE is associated with an individual's perception of the technology's usefulness in the performance enhancement [28,47,48]. In developing countries like Vietnam, the readiness of infrastructure is an important condition for press agencies and journalists to apply a new technology like BC. On that basis, the hypothesis here is:

H5 TRE has a positive influence on BIN of applying BC in the field of Journalism in Vietnam.

*Trust* (TRU) is a belief that has been confirmed to positively influence intended behavior to adopt a technology [29,49,50]. The adoption of BC still has many obstacles; however, its potential is quite great for developing the media industry [6]. As a result, before the barriers to the adoption of BC are removed, the belief of individuals and organizations in this new technology is a prerequisite for its future adoption. The hypothesis here is:

H6 TRU has a positive influence on BIN of applying BC in the field of Journalism in Vietnam.

*Regulatory Support* (RSU) is an essential factor that should be considered in adopting new technology, such as BC in organizations and businesses in different countries [28]. Legal support and related regulations might ease the adoption of a technology [40]. In Vietnam, the press is a specific field; press agencies are under the management of the government as in other countries that exercise the top-down management approach [51]; therefore, the adoption of BC in press activities in Vietnam requires the government's support. On that basis, we propose the following hypotheses.

H7 RSU has a positive influence on BIN of applying BC in the field of Journalism in Vietnam.

H8 RSU regulates the impact of FCO on BIN of applying BC in the field of Journalism in Vietnam.

H9 RSU regulates the impact of TRE on BIN of applying BC in Journalism in Vietnam.

## 4 Methods

### 4.1 Data collection and procedures

This study was approved by the Academic Committee at University of Sciences, Thai Nguyen University, Thai Nguyen, Vietnam. Data were collected from December 2021 to March 2022 through a questionnaire written in Vietnamese with the respondents' voluntary consent. To get a suitable assessment, we first employed convenience sampling and purposeful sampling techniques to select 287 journalists and officials (see Table 1) in six major news agencies in Vietnam who were aware of BC technology. With the approvals of the boards of directors of these agencies, we sent an invitation email to all journalists and officials. Then, we used the purposeful sampling technique to select those journalists and officials who were aware of BC technology and volunteered to answer the questionnaire.

**Table 1:** Respondents' information.

		Frequency	Percent
Gender	Male	191	66.6
	Female	96	33.4
Age	22–29 Years Old	100	34.8
	30–39 Years Old	119	41.5
	40–49 Years Old	53	18.5
	50 and above	15	5.2
Length of time with organisation	Less than a year	15	5.2
	1–2 years	79	27.5
	3–5 years	103	35.9
	6–10 years	77	26.8
	Above 10 years	13	4.5
Primary Job Scope	Journalist	114	39.7
	Reporter	104	36.2
	Technician	13	4.5
	Office staff	28	9.8
	Editor	15	5.2
	Managers	13	4.5
What is your role in the adoption of new technology in the press?	I participate in decision-making to use new technology	30	10.5
	I participate in the process of recommending new technology	72	25.1
	I participate in both processes above	40	13.9
	I have no role	145	50.5
What is your opinion on the adoption of blockchain in journalism in Vietnam today?	Not applicable in Vietnam	35	12.2
	Unable to apply immediately	161	56.1
	Applicable within the next one year	6	2.1
	Maybe in the future	82	28.6
	We are applying Blockchain in the agency	3	1.0

We considered the sample size recommendations in Partial Least Squares SEM (PLS-SEM) built on the properties of OLS regression. Accordingly, when the measurement and structural models include seven independent variables, 188 observations are required to achieve an 80% statistical power to detect R^2^ values of at least 0.1 with a 1% probability of error [52], p. 48. In addition, the sample size for this investigation was calculated using G*Power version 3.1 software [53]. A sample size of 198 is suggested for an effect size of f^2^ = 0.15, a probability of error of = 0.01, a power level of (1 − β) = 0.95, and a number of predictors of 7. The presented statistics were satisfactory, and the sample size was adequate.

In this study, most respondents were authorized journalists (39.7%), followed by reporters (36.2%) and officials. Most of them worked for 3–5 years (35.9%) and 6–10 years (26.8%), 1–2 years (27.5%), and at least over ten years (4.5%). Most of the respondents were under 40 years old, of which those in the age range of 22–29 accounted for 34.8%, and those from 30 to 39 years old accounted for 41.5%. Male respondents still made up for 66.6%. More than half of the respondents had no role in the adoption of new technology at the agency, and the slightly over a quarter (25.1%) involved in the proposal process; a small percentage (10.5%) was involved in the decision-making process and both processes above (13.9%).

The original questionnaire was mainly based on the scales assessing the perception of BC in journalism [4,5,7,8,12], and the scales of factors affecting the adoption of new technology [28,29,33,37,54]. We first piloted the original questionnaire with 30 participants (10 researchers, five experts, and 15 experienced journalists) to check its validity. We then used the participants' opinions to revise the questionnaire. The official questionnaire consisted of two main parts: participants’ demographic information and 34 items (see Appendix A1) on a Likert scale of 1–5 (from 1 = strongly disagree to 5 = strongly agree).

### 4.2 Data analysis

SPSS version 26.0 software (IBM) was utilized to analyze the survey data gathered. We used the covariance matrix with the PLS-SEM, and precisely SmartPLS 4 to analyze the data collected, test the research hypotheses [52,55,56]. The research hypotheses were tested through two steps: (1) Evaluation of the measurement model and (2) Evaluation of the structural model. To evaluate the measurement model, the researcher first, to evaluate the convergence validity on Smartpls, afterward, we checked the common method bias (CMB) about whether or not the research model was inflated through Harman's single factor test using SPSS software (IBM). To evaluate the discriminant, we evaluated discriminant by using the traditional approach by Ref. [57] and Heterotrait-Monontrait Ratio (HTMT) [58]. First, we examined the variance inflation factor (VIF) to evaluate the structural model's presence of strongly correlated constructs. Second, we used Stone- Q^2^ Geisser's test to determine the model's predictive capability.

## 5 Result

### 5.1 Measurement model

We examined the average variance extracted (AVE). The results of convergence validity on Smartpls showed that the AVE index was greater than the recommended value of 0.5, indicating that the scales were all convergent [52,55,59]. Then, we examined the reliability of the scale (Cronbach's alpha, composite reliability, and Dijkstra-Henseler's rho_A). The results showed that Cronbach's alpha and Composite reliability were both greater than 0.7 (see Table 2), and the rho_A was greater than 0.7 [52,60,61].

**Table 2:** Values of the scale.

	Mean	Std. Deviation	Cronbach's Alpha	rho_A	Composite Reliability	Average Variance Extracted (AVE)	Inner Variance Inflation Factor (VIF) Values
**PEX**	**3.23**	**.742**	**0.788**	**0.804**	**0.875**	**0.7**	**1.27**
Q1	2.30	.844					
Q4	3.93	.964					
Q15	3.48	.848					
**EEX**	**3.39**	**.773**	**0.822**	**0.827**	**0.882**	**0.65**	**1.325**
Q2	4.02	1.007					
Q7	4.03	1.005					
Q20	2.19	.996					
Q33	3.33	.822					
**FCO**	**2.79**	**.736**	**0.841**	**0.842**	**0.893**	**0.677**	**1.350**
Q11	3.28	.852					
Q21	3.31	.818					
Q26	2.18	.982					
Q29	2.40	.929					
**TRE**	**2.90**	**.754**	**0.834**	**0.834**	**0.889**	**0.668**	**1.321**
Q5	2.30	.965					
Q22	3.32	.879					
Q30	2.53	.938					
Q34	3.47	.911					
**TRU**	**3.68**	**.788**	**0.858**	**0.870**	**0.903**	**0.701**	**1.464**
Q3	3.75	.980					
Q8	4.03	.998					
Q27	3.55	.963					
Q32	3.40	.825					
**TAF**	**3.80**	**.723**	**0.833**	**0.836**	**0.889**	**0.666**	**1.433**
Q6	4.19	.896					
Q12	4.07	.999					
Q16	3.45	.822					
Q25	3.48	.831					
**RSU**	**3.67**	**.708**	**0.818**	**0.845**	**0.878**	**0.643**	**1.037**
Q9	3.98	.902					
Q13	3.90	.893					
Q19	3.38	.872					
Q23	3.33	.797					
**BIN**	**3.61**	**.579**	**0.857**	**0.859**	**0.903**	**0.7**	
Q14	3.17	.679					
Q17	4.03	.701					
Q28	3.22	.686					
Q31	4.03	.701					

The common method bias (CMB) test findings suggested that the first component accounted for 22,325% of the variance in the data. Since the result was below the 50% criterion, it was possible to conclude that there was no CMB issue in our research data [28,29,60]. In addition, the correlations were less than 0.90 (see Table 3), indicating that CMB did not pose a significant threat to the interpretation of our study [28,62]. In order to test whether or not the observed variables were significant in the model, we used the value of the outer loading (see Appendix A2) to evaluate. The result showed that items Q10, Q18, and Q24 should have been removed because their outer loadings were smaller than 0.7 [52,63,64].

**Table 3:** Fornell-Larcker criterion.

	BIN	EEX	FCO	PEX	RSU	TAF	TRE	TRU
BIN	**0.837**							
EEX	0.469	**0.807**						
FCO	0.542	0.354	**0.823**					
PEX	0.282	0.414	0.298	**0.837**				
RSU	0.141	0.03	0.008	0.025	**0.802**			
TAF	0.581	0.16	0.266	0.105	−0.03	**0.816**		
TRE	0.584	0.155	0.333	0.179	0.037	0.362	**0.817**	
TRU	0.419	0.072	0.303	0.167	−0.073	0.484	0.379	**0.837**

The square root of AVE (the top value of each column) from the discriminant test was larger than the correlations between latent variables (correlation coefficient is below the first value of the column) (see Table 3), so discriminant validity was guaranteed [52,57]. The discriminant validity was guaranteed because the HTMT values (see Appendix A3) were smaller than 0.85 [52,58].

### 5.2 Structural model

The results of the VIF value in Table 2 show that the VIF coefficients are all less than 3. Thus, this research model had no multicollinearity phenomenon [65]. Q^2^ values (=0.465) indicated that the model had a higher predictive potential (see Table 4), and the PLS-path model had a moderate to sizeable predictive relevance [65]. The holdout test's Q^2^ values (= 0.653) value from PLSpredict test validated the prediction model's robustness [29,52,66].

**Table 4:** Construct cross-validated redundancy and PLS Predict test.

Blindfolding			
Latent variable	Sum of squares of observations (SSO)	Sum of squares of prediction errors (SSE)	Q^2^ (=1-SSE/SSO)
BIN	1148	613.68	0.465
PLSpredict			
Latent variable	Root mean squared error (RMSE)	Mean absolute error (MAE)	Q^2^_predict
BIN	0.594	0.409	0.653

**Fig. 4:**
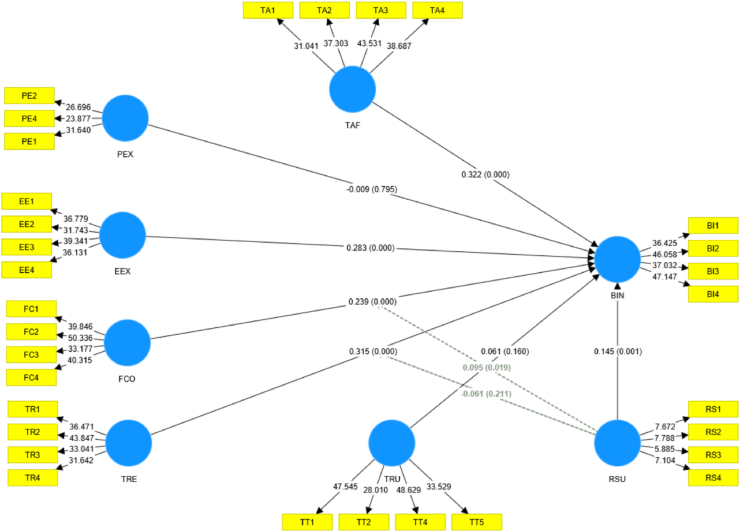
Structural model.

The structural model examination shows the results of the hypothesis testing together with their associated values: *p*-values and *t*-values (see Table 5; Fig. 4). The t-values were larger than 1.96, indicating that the relationship was statistically significant at the 95% confidence level [28]. The results showed that TAF was positively associated with BIN (β = 0.322, *p* < 0.01), confirming H1. Additionally, EEX was strongly associated with BIN (β = 0.283, p < 0.01), corroborating H3. Similarly, FCO was found to be positively related to BIN (β = 0.239, *p* < 0.01), validating H4. Furthermore, TRE was positively associated with BIN (β = 0.315, *p* < 0.01), confirming H5. Additionally, RSU was positively correlated with BIN (β = 0.145, *p* < 0.01). As a result, H7 was validated. Also, RSU modified the connection between FCO and BIN positively (β = 0.095, *p* < 0.05). As a result, H8 was supported.

**Table 5:** The outcome of the structural model examination.

Hypotheses	Patch	Coefficient/Beta-values (*β*)	*t*-values	*p*-Values	Decision
H1	TAF -> BIN	0.322	7.353	0.000	Supported
H2	PEX → BIN	−0.009	0.258	0.795	Not supported
H3	EEX → BIN	0.283	7.489	0.000	Supported
H4	FCO → BIN	0.239	5.858	0.000	Supported
H5	TRE → BIN	0.315	7.871	0.000	Supported
H6	TRU → BIN	0.061	1.375	0.160	Not supported
H7	RSU → BIN	0.145	3.305	0.001	Supported
H8	RSU*FCO → BIN	0.095	2.359	0.019	Supported
H9	RSU*TRE → BIN	−0.061	1.258	0.211	Not supported

However, the relationship between PEX (β = −0.009, p > 0.05) and TRU (β = 0.061, p > 0.05) with BIN was statistically insignificant and hence unsupported by the results of this study. As a result, H2 and H6 were not validated. Unlike expected, RSU did not affect the link between TRE and BIN (β = −0.061, *p* > 0.05). As such, H9 was disallowed.

The results showed that the endogenous variables were relatively accurate (R^2^ = 0.685). This indicated that the seven exogenous factors provided a moderate explanation of the variance in BIN and were considered significant for interpretation [28,52]. This study examined effect size based on Cohen f^2^ values (see Table 6). PEX and TRU had f^2^ values of very small size, while other variables had f^2^ values ranging from small to medium size [67,68].

**Table 6:** Effect size (f^2^).

	TAF	PEX	EEX	FCO	TRE	TRU	RSU
BIN	0.23	0.000	0.192	0.134	0.238	0.008	0.065

Note: BIN = Behavioral Intention; TAF = Technology affinity; PEX = Performance Expectancy; EEX = Effort Expectancy; FCO = Facilitating Condition; TRE = Technology Readiness; TRU = Trust; RSU = Regulatory Support.

## 6 Discussion

Several proposed models for how blockchain technology could be used in journalism to achieve decentralization, share information, personalize agenda settings, collect public news, and authenticate news articles suggest that blockchain technology may be a viable solution for journalism (e.g., Refs. [1,5,69]). Agrawal et al. [1] argue that blockchain could enhance the veracity of news items. Le and Loebbecke [69] believe that blockchain might be used to improve the monetization of online news. Kim and Yoon [5] suggest blockchain could improve the delivery of articles and the collection of public opinion in journalism. Consequently, blockchain technology promisingly brings about a variety of benefits in the field of journalism.

This study sought to empirically investigate the determinants of BC adoption in journalism in Vietnam by using an extended UTAUT model [26,28,29,33]. Unlike previous studies, this study included such factors as the impact of RSU on BIN. The results show that the proposed model had a good explanatory meaning (R^2^ = 0.685) for the intention of applying BC in journalism activities in Vietnam.

This study demonstrates that TAF, EEX, FCO, TRE, and RSU significantly impacted BIN. This finding confirmed the previous studies by Franke, T., C. et al. (2019) and [28]. This finding indicates that TAF and EEX with technology would/could facilitate the adoption of new technologies to professional operations [26,28,29,36,37,60]. Meanwhile, organizational and managerial elements, such as FCO, TRE, and RSU, will ensure that agency personnel were fully informed of and enthusiastic about using a new technology [26,28,29,33,40,70]. These results imply that to apply BC technology in practical activities at the agency, it is necessary to have policy support from the management and leadership levels to ensure the infrastructure and the technical layer are suitable for applying new technology. In addition, it is also necessary to foster skills to improve the ability of employees to use new technology in the agency.

We could not detect the effect of PEX and TRU on BIN. However, this result was in line with previous research in different disciplines worldwide [29,33,45,46,49,50]. As [29] explained, cultural and socioeconomic differences between nations may contribute to these disparate research outcomes. At the time of this study, the development of BC in Vietnam was still in its infancy. In addition, only 56.1% of respondents were unable to apply BC in Vietnam immediately, and 12.2% believed that the technology was not applicable, resulting in a loss of confidence and reasonable expectations of BC adoption. Therefore, it is vital first to enhance the staffs’ understanding of BC technology and its potential in journalism. Employees can learn about the qualities and uses of new technology through training, documentation, books, and newspapers.

The current study also found the regulatory effect of RSU on the relationship between FCO and BIN, and that RSU did not affect the link between TRE and BIN, which confirmed the study by Ref. [28] in Malaysia. The implication is that agencies seeking to adopt must handle a wide range of issues affecting Vietnam journalism management stakeholders and state policy issues with immature technology. The findings have ramifications for news organizations that wish to implement new technologies, such as BC, in Vietnam and other developing countries. They might need to arrange the essential support circumstances, including legislation, technological infrastructure, and resources suited to the new technology. Next, news agencies may cultivate and enhance the qualifications of their employees, including their awareness of and proficiency with new technology access, so that they may confidently and competently use new technology [4,6,8].

This study has several contributions. Theoretically, it introduced a model to comprehend the purpose of implementing new journalism technologies. This is the first attempt to employ the PLS-SEM model in data analysis in the field. This study contributes to the literature on technology acceptability by testing a unified model, bridging the gap between the literature on BC technology adoption in journalism and media in Vietnam and other countries with similar contexts.

## 7 Conclusion

This study provided and discussed the factors that affected the intention to apply BC in journalism in Vietnam. The current study provides actual evidence of the factors influencing BC adoption in journalism. The research results give suggestions and concerns about when using BC in journalism in Vietnam and other countries with similar situations.

This endeavor has added multiple insights, conducted in a context where residents and staffs in the field of journalism are familiarized themselves with blockchain technology. Theoretically, it offers a framework for understanding blockchain's value as an innovative journalism technology. Although involving 287 participants in understanding journalists and officials, this sample was representative of those familiar with blockchain technology in journalism. A comprehensive survey of the literature has provided an overview of the utilization of blockchain technology and contextual factors that have impacted the acceptance of this technology, which sets in-depth foundations for arguments for the theoretical framework and interpretation of the results.

There are several limitations of this study. First, the scarcity of empirical studies regarding the adoption of BC in journalism limits the comparison of results. Further studies can retest the hypotheses in different contexts to provide more insights into the potential adoption of BC technology. Another limitation is generalizability. As this study was conducted in a country with developing journalism, it was hard to compare with previous research in the field. The experience of BC implementation in Vietnam is still in its infancy in most fields, which affects the collection of research samples. News agencies and their employees hesitated to participate in the study because they did not fully understand the applicability of BC in their area. Future studies should consider selecting a larger sample size based on specific target groups to compare their perceptions and attitudes regarding the use of new technologies. Longitudinal research results may also help examine changes in people's perceptions of the adoption.

## Figures and Tables

**Table:** 

Construct	Measurement item	Item	Source
Performance Expectancy	Blockchain adoption can help me access information faster and more accurately	Q1	Derived from concerns in [4,5,7,8,12]
	Blockchain adoption helps me to ensure information copyright and avoid unauthorized copying of information	Q4	
	Blockchain adoption helps me save time verifying information, avoiding fake news	Q10	
	Blockchain adoption helps press news content have higher trust value	Q15	
	Blockchain adoption will promote a more efficient pay newspaper model	Q18	
Effort Expectancy	Investment cost is the main concern for Blockchain adoption in my office	Q2	Adapted from Tai and Ku, 2013, [28]
	The support from the management board plays an important role for the application of Blockchain in my organization	Q7	
	I have the skills to apply Blockchain in my professional activities	Q20	
	My organization can afford to apply Blockchain	Q33	
Facilitating Conditions	My organization has enough resources to apply Blockchain	Q11	Adapted from [28,33]
	My organization has a technical support system ready for Blockchain adoption	Q21	
	My organization have necessary knowledge to apply Blockchain	Q26	
	The leaders of my organization show interest in Blockchain	Q29	
Technology Readiness	My organization details the potential of Blockchain adoption	Q5	Adapted from [28,47,48]
	My organization's technical infrastructure is suitable for Blockchain adoption	Q22	
	Blockchain is in the orientation of applying modern technology in my organization	Q30	
	My organization's security system is suitable for Blockchain adoption	Q34	
Trust	I believe in the feasibility of applying Blockchain in journalism	Q3	Revised from [28,29,50,54]
	I believe Blockchain can help secure data and limit fraud	Q8	
	I believe I can master Blockchain	Q24	
	I am willing to apply Blockchain in my professional activities	Q27	
	I believe Blockchain will make my work more efficient	Q32	
Technology Affinity	I am proficient in using computer technology	Q6	Adapted from Franke et al., 2017, [28,37]
	I love experimenting with new technologies	Q12	
	I don't mind spending time to get used to new technical technology	Q16	
	It doesn't take me too long to master new technology	Q25	
Regulatory Support	My organization's decision to apply Blockchain depends on common standards in journalism in Vietnam	Q9	Derived from concerns in Refs. [7,51], Paul et al., 2019, [5,8]
	The guidelines and policies of the management levels determine the possibilty to apply Blockchain at the agency	Q13	
	Regulations on information security, prevention of fake news and false information will affect the process of applying Blockchain at press agencies.	Q19	
	The change in the pay newspaper reading model affects the adoption of Blockchain in news agencies	Q23	
Behavioral Intention	I already intend to apply Blockchain in the future	Q14	Adapted from [26,28,29]
	I think my organization will adopt Blockchain in the future	Q17	
	My organization will be one of the pioneers in applying Blockchain	Q28	
	My agency regularly applies new technology in professional activities	Q31	

**Table:** 

	BIN	EEX	FCO	PEX	RSU	TAF	TRE	TRU
Q14	0.812							
Q17	0.851							
Q28	0.831							
Q31	0.852							
Q2		0.810						
Q7		0.798						
Q20		0.799						
Q33		0.819						
Q11			0.823					
Q21			0.853					
Q26			0.799					
Q29			0.816					
Q1				0.862				
Q4				0.820				
Q15				0.828				
Q9					0.783			
Q13					0.828			
Q19					0.785			
Q23					0.811			
Q6						0.800		
Q12						0.813		
Q16						0.835		
Q25						0.816		
Q5							0.806	
Q22							0.830	
Q30							0.818	
Q34							0.815	
Q3								0.867
Q8								0.795
Q27								0.869
Q32								0.816

**Table:** 

	BIN	EEX	FCO	PEX	RSU	TAF	TRE	TRU
BIN								
EEX	0.553							
FCO	0.639	0.425						
PEX	0.340	0.501	0.364					
RSU	0.163	0.053	0.055	0.084				
TAF	0.682	0.188	0.312	0.129	0.079			
TRE	0.688	0.184	0.397	0.221	0.087	0.435		
TRU	0.484	0.092	0.355	0.204	0.100	0.564	0.444	
