# Supplementary material for: Determinants of blockchain adoption in news media platforms: A perspective from the Vietnamese press industry
Source: Heliyon. 2022 Dec 30;9(1):e12747. doi: 10.1016/j.heliyon.2022.e12747 (PMC9849998; doi:10.1016/j.heliyon.2022.e12747)
Supplement: Multimedia component 1 [file mmc1.docx]

**QUESTIONNAIRE**

This study is on the determinant of Blockchain adoption in news media platforms with a perspective from the Vietnamese press industry. As your answers are very important to the success of the study, please provide responses as accurately as possible. Your answers will be kept confidential. Your participation in this study is voluntary. You can refuse to answer the following questions.

**Personal information**

Gender:

1. Male

2. Female

Age:

1. 22 - 29 Years Old

2. 30 - 39 Years Old

3. 40- 49 Years Old

4. 50 and above

Length of time with organisation:

1. Less than a year

2. 1 – 2 years

3. 3 – 5 years

4. 6 – 10 years

5. Above 10 years

Primary Job Scope:

1. Journalist

2. Reporter

3. Technician

4. Office staff

5. Editor

6. Managers

What is your role in the adoption of new technology in the press?

1. I participate in decision-making to use new technology

2. I participate in the process of recommending new technology

3. I participate in both processes above

4. I have no role

What is your opinion on the adoption of blockchain in journalism in Vietnam today?

1. Not applicable in Vietnam

2. Unable to apply immediately

3. Applicable within the next one year

4. Maybe in the future

5. We are applying Blockchain in the agency

**Please make ONLY ONE CHOICE:**

1=absolutely disagree, 2=disagree, 3=neither agree or disagree, 4=agree, 5=absolutely agree

| Question | Statement | Rating | | | | |
| --- | --- | --- | --- | --- | --- | --- |
|  |  | 1 | 2 | 3 | 4 | 5 |
| 1 | Blockchain adoption can help me access information faster and more accurately |  |  |  |  |  |
| 2 | Investment cost is the main concern for Blockchain adoption in my office |  |  |  |  |  |
| 3 | I believe in the feasibility of applying Blockchain in journalism |  |  |  |  |  |
| 4 | Blockchain adoption helps me to ensure information copyright and avoid unauthorized copying of information |  |  |  |  |  |
| 5 | My organization details the potential of Blockchain adoption |  |  |  |  |  |
| 6 | I am proficient in using computer technology |  |  |  |  |  |
| 7 | The support from the management board plays an important role for the application of Blockchain in my organization |  |  |  |  |  |
| 8 | I believe Blockchain can help secure data and limit fraud |  |  |  |  |  |
| 9 | My organization's decision to apply Blockchain depends on common standards in journalism in Vietnam |  |  |  |  |  |
| 10 | Blockchain adoption helps me save time verifying information, avoiding fake news |  |  |  |  |  |
| 11 | My organization has enough resources to apply Blockchain |  |  |  |  |  |
| 12 | I love experimenting with new technologies |  |  |  |  |  |
| 13 | The guidelines and policies of the management levels determine the possibilty to apply Blockchain at the agency |  |  |  |  |  |
| 14 | I already intend to apply Blockchain in the future |  |  |  |  |  |
| 15 | Blockchain adoption helps press news content have higher trust value |  |  |  |  |  |
| 16 | I don't mind spending time to get used to new technical technology |  |  |  |  |  |
| 17 | I think my organization will adopt Blockchain in the future |  |  |  |  |  |
| 18 | Blockchain adoption will promote a more efficient pay newspaper model |  |  |  |  |  |
| 19 | Regulations on information security, prevention of fake news and false information will affect the process of applying Blockchain at press agencies. |  |  |  |  |  |
| 20 | I have the skills to apply Blockchain in my professional activities |  |  |  |  |  |
| 21 | My organization has a technical support system ready for Blockchain adoption |  |  |  |  |  |
| 22 | My organization's technical infrastructure is suitable for Blockchain adoption |  |  |  |  |  |
| 23 | The change in the pay newspaper reading model affects the adoption of Blockchain in news agencies |  |  |  |  |  |
| 24 | I believe I can master Blockchain |  |  |  |  |  |
| 25 | It doesn't take me too long to master new technology |  |  |  |  |  |
| 26 | My organization have necessary knowledge to apply Blockchain |  |  |  |  |  |
| 27 | I am willing to apply Blockchain in my professional activities |  |  |  |  |  |
| 28 | My organization will be one of the pioneers in applying Blockchain |  |  |  |  |  |
| 29 | The leaders of my organization show interest in Blockchain |  |  |  |  |  |
| 30 | Blockchain is in the orientation of applying modern technology in my organization |  |  |  |  |  |
| 31 | My agency regularly applies new technology in professional activities |  |  |  |  |  |
| 32 | I believe Blockchain will make my work more efficient |  |  |  |  |  |
| 33 | My organization can afford to apply Blockchain |  |  |  |  |  |
| 34 | My organization's security system is suitable for Blockchain adoption |  |  |  |  |  |
